# Factors Associated with Health Check-up and Cancer Screening Participation among Family Caregivers of Patients with Dementia: A Cross-Sectional Study

**DOI:** 10.1186/s12889-021-11768-8

**Published:** 2021-09-26

**Authors:** Bomgyeol Kim, Yejin Lee, Jin-Won Noh, Tae Hyun Kim

**Affiliations:** 1 Department of Public Health, Graduate School, Yonsei University, 50-1 Yonsei-ro, Seodaemun-gu, Seoul, 03722 Republic of Korea; 2 Department of Public Health, Korea University College of Medicine, 73 Inchon-ro, Seongbuk-gu, Seoul, 02841 Republic of Korea; 3 Division of Health Administration, College of Software and Digital Healthcare Convergence, Yonsei University, 1 Yeonsedae-gil, Wonju-si, Gangwon-do Republic of Korea; 4 Department of Healthcare Management, Graduate School of Public Health, Yonsei University, 50-1 Yonsei-ro, Seodaemun-gu, Seoul, 03722 Republic of Korea

**Keywords:** Health check-up, Cancer screening, Informal caregivers, Dementia care

## Abstract

**Background:**

Providing care for patients with dementia can negatively influence the physical health and health behaviours of family caregivers. A better understanding of the factors associated with health check-up and cancer screening participation is vital for developing effective interventions. Thus, this study aimed to identify factors associated with health check-up and cancer screening participation among family caregivers of patients with dementia.

**Methods:**

This was a cross-sectional study that analysed the data of 2,414 family caregivers of patients with dementia collected by the Korea Community Health Survey in 2017. A binomial logistic regression analysis was performed to identify demographic, socioeconomic, and health status factors associated with health check-up and cancer screening participation among family caregivers of patients with dementia.

**Results:**

Health check-up and cancer screening rates among family caregivers of patients with dementia were 68.7% and 61.4%, respectively, which were significantly lower than the rates for individuals who were not caregivers of patients with dementia. Those with lower education levels had lower odds ratios (OR) for both health check-up (OR: 0.60) and cancer screening (OR: 0.59) participation. In addition, symptoms of depression were associated with lower participation (health check-up OR: 0.67; cancer screening OR: 0.65).

**Conclusions:**

More targeted disease prevention and management strategies must be developed for family caregivers of patients with dementia, particularly those with depressive symptoms and lower education levels.

## Background

Dementia affects 10.16% of older Korean adults. Despite significant medical advances, dementia is not curable and poses a significant burden on patients and their families [1, 2]. Many national health policies focus on effective and efficient ways to enable patients with dementia to live as independently as possible in their communities [3]. Prior studies have shown that family caregivers play an important role in maintaining the lives of patients with dementia in their communities [4, 5]. In this study, a family caregiver or informal caregiver is defined as a family member who voluntarily assists an individual with an acute or chronic condition with several tasks, such as bathing, dressing, and taking medications [6, 7]. Family caregivers of patients with dementia experience stress, burnout, depression, and socioeconomic difficulties caused by prolonged caregiving [8]. Consequently, they are considered ‘secondary patients’ or ‘hidden patients’ and caring for older adults with dementia in the home setting is believed to be more burdensome than caring for a terminal cancer patient [9, 10]. Since informal care often negatively impacts the health of family members providing care, the need to pay attention to the health behaviours of this population is essential [11].

As dementia progresses, the need for care increases significantly, and professional services are often required to support not only the family’s health and welfare to supplement their care but also their activities [3]. Families providing care risk developing further health complications themselves [6, 7]. Thus, their health must be checked routinely, and abnormalities must be treated at the national level to promote the healthy life of families. Health check-ups and cancer screenings are strategies for the early detection, treatment, and management of diseases to prevent premature death or severe complications [12, 13]. Through health check-up and cancer screening participation, preventive treatment services can result in lower potential medical costs and the early treatment of diseases using relatively simple methods and yielding good outcomes [14]. The government strongly recommends regular health check-ups to identify diseases in a timely manner [15]. Thus, health check-ups and cancer screenings are effective and essential health behaviours for detecting diseases early [16]. However, because of the prolonged burden of informal care, those who live family members with dementia may not attend health check-ups and cancer screenings. Furthermore, family caregivers overlook community resources, do not access available resources, and are not aware of the best methods for using the resources that are available [17]. Accordingly, they often neglect their health care needs while caring for a family member with an illness, leading to the deterioration of their health and well-being [18–20]. Increasing the rates of health check-ups among families providing informal care to patients with dementia is critical for enhancing their health and quality of life. Therefore, examining health check-up rates and identifying factors associated with health check-up and cancer screening participation is important to promote informal caregivers’ effective participation in these health behaviours.

Previous studies have identified several factors associated with health check-up rates among Korean adults, which include sex, age, area of residence, marital status, education level, type of health insurance, household income, subjective health status, chronic disease, depression, smoking, drinking, and regular exercise [21–23]. Factors related to cancer screenings included sex, age, area of residence, type of health insurance, private health insurance, monthly household income, chronic diseases, health check-up intention, concerns about the risk of cancer, history of cancer screening, and regular exercise [13, 24, 25]. However, studies examining caregiver health behaviours in Korea are lacking, particularly the rates of health check-up and cancer screening participation in family caregivers of patients with dementia. Therefore, examining whether these factors influence families of patients with dementia equally is necessary.

Thus, considering the growing number of patients with dementia and the consequent rise in the caregiving burden placed on their families, this study aimed to identify factors associated with health check-up and cancer screening participation among family caregivers of patients with dementia. The study’s objectives were as follows: 1) to compare and analyse health check-up and cancer screening rates between family caregivers of patients with dementia and individuals who were not caregivers of patients with dementia; 2) to identify whether and how health check-up and cancer screening rates differ according to demographic, socioeconomic, and health status factors; and 3) to determine the factors associated with health check-up and cancer screening rates of family caregivers of patients with dementia.

## Methods

### Study design

We used a cross-sectional study to identify factors associated with health check-up and cancer screening participation rates among family caregivers of patients with dementia. Cross-sectional studies are especially appropriate for understanding phenomena and explaining relationships between phenomena at a specific point in time [26].

### Data and study population

This study used raw data from the 2017 Korea Community Health Survey (KCHS) conducted by the Korea Centres for Disease Control and Prevention. The KCHS is a general survey approved by Statistics Korea (approval number: 117075) conducted per Article 4 (Community Health Survey) of the Regional Public Health Act and Article 2 (Method and Content of the Community Health Survey) of the Enforcement Decree of the same act [27]. After selecting a sample plot through probability proportional to size sampling in tong, ban, and ri units (referring to the administrative districts in Korea), households included in the survey were selected through systematic sampling. Trained examiners visited each selected household and conducted face-to-face interview surveys using computer-assisted personal interviewing [27]. Data from the KCHS are open source and can be used as primary data through the KCHS website after submitting a data usage plan. Therefore, ethical approval was not required for this study. Accordingly, this study did not obtain informed consent from the participants because their data was fully anonymised before the analysis.

Of the total 228,381 respondents, we included the 2,414 family caregivers of patients with dementia who answered ‘yes’ to the following question: ‘Does your household currently include a patient diagnosed with dementia by a physician’?

### Measurements

#### Dependent Variables

The dependent variables in this study were health check-up and cancer screening participation. Health check-up participation was assessed using the question ‘Have you attended a health check-up (excluding cancer screenings) in the past two years to check your health status unprompted by any particular health problems’? The health check-ups include the national general health check-ups (conducted every other year), examinations and counselling, physical measurements, blood pressure measurements, chest radiography, and blood tests. In addition to the national health check-ups, any check-up performed according to a patient’s individual needs were included. Those who answered ‘no’ to the question were classified into the group without health check-ups, and those who answered ‘yes’ were classified into the group with health check-ups.

Cancer screening participation was also assessed using the following question: ‘Have you undergone a cancer screening in the past two years to check your health status unprompted by any particular health problems’? Cancer screening included national cancer screening for stomach, liver, colon, breast, cervical, and lung cancer; screening conducted according to individual needs; and those provided by the government. Those who answered ‘no’ to the question were classified into the group with no cancer screening, and those who answered ‘yes’ were classified into the group with cancer screening.

#### Independent Variables

The following demographic factors were assessed: sex, age, and marital status. Age was divided into those aged 19–44 years, 45–64 years, and ≥ 65 years. Marital status was classified as single, married, and other (divorced, widowed, or separated) using the marital status question item. The area of residence was classified into capital (Seoul Special City, Incheon Metropolitan City, and Gyeonggi Province) and non-capital (all other cities and provinces).

Furthermore, we assessed socioeconomic factors, including education level, occupation category, and income level. Education level was classified into ≤ elementary school graduates, middle school graduates, high school graduates, and ≥ college graduates according to the highest level of education completed. The occupation category was classified into white-collar (managers, professionals and relevant workers, and office workers), sales and service (service workers, sales associates), blue-collar (agricultural, forestry and fishery workers, technicians and relevant technical workers, device, machine manipulation and assembly workers, and elementary workers), and others (students, housewives, and unemployed). Income level was classified into < 2 million KRW, 2–4 million KRW, and > 4 million KRW.

Health status factors, subjective health status, stress levels, symptoms of depression, and chronic diseases were assessed. The subjective health status and stress levels were self-reported by the subjects in a table. The subjective health status was classified as good (very good and good), moderate (moderate), or poor (poor and very poor). Subjective stress levels were assessed based on daily levels of stress and classified as very high, high, low, and very low. Depressive symptoms were considered to be absent for mild depression (≤9 points) and to be present for severe depression (> 9 points) using the Patient Health Questionnaire (PHQ)-9. The PHQ-9 is a tool for assessing for depressive symptoms and monitoring patients’ treatment response [28]. Chronic disease was considered present for a person who responded ‘yes’ to one or more of the following seven diseases: hypertension, diabetes mellitus, dyslipidaemia, stroke, myocardial infarction, arthritis, and cataracts, which were surveyed as chronic diseases in the KCHS data.

### Statistical analysis

The general characteristics and distributions of each factor were analysed using frequency analyses, and the data were presented as counts and percentages. We used the chi-square test to calculate the distribution of patient characteristics according to health check-up and cancer screening participation. This test is commonly used to assess the association between two or more categorical variables. Factors associated with health check-ups and cancer screening participation were identified using binomial logistic regression analyses. The results were reported using odds ratios (ORs) and confidence intervals (CIs). Differences were considered statistically significant at *p <* 0.05. All statistical analyses were performed using the SPSS Statistics software (version 22.0, IBM, Chicago, IL, USA).

## Results

### Health check-up and cancer screening participation among family caregivers of patients with dementia compared to that of individuals who were not caregivers of patients with dementia

Table 1 shows the health check-up and cancer screening participation among family caregivers of patients with dementia compared to that of individuals who were not caregivers of patients with dementia. The health check-up rate of family caregivers of patients with dementia was 68.7% and their cancer screening rate was 61.4%. In contrast, the health check-up rate of individuals who were not caregivers of patients with dementia was 73.0%, with a cancer screening rate of 63.4%. Family caregivers of patients with dementia and individuals who were not caregivers of patients with dementia significantly differed in terms of health check-up (*p <* 0.001) and cancer screening participation (*p =* 0.038).

**Table 1:** Health check-up and cancer screening participation of caregivers of patients with dementia compared to that of individuals who were not caregivers of patients with dementia

Variable	Health check-ups			Cancer screening		
	Yes	No	***P***-value	Yes	No	***P***-value
	n (%)	n (%)		n (%)	n (%)	
**Family caregivers of patients with dementia (*n=*2414)**	1658 (68.7)	756 (31.3)	<0.001	1482 (61.4)	932 (38.6)	0.038
**Individuals who were not caregivers of patients with dementia (*n=*221451)**	161638 (73.0)	59813 (27.0)		140485 (63.4)	80966 (36.6)	

### Differences in health check-up and cancer screening participation according to general characteristics

Table 2 shows the differences in health check-up and cancer screening participation according to participants’ general characteristics. Health check-up participation significantly differed according to age, marital status, area of residence, education level, occupation category, income level, symptoms of depression, and chronic disease. Cancer screening participation significantly differed according to sex, age, marital status, area of residence, occupation category, symptoms of depression, and chronic disease.

**Table 2:** Differences in health check-up and cancer screening participation according to general characteristics

Variable	Total (***N=***2414)	Health check-up participation			Cancer screening participation		
		No (***n=***756)	Yes (***n=***1658)	***P-***value	No (***n=***932)	Yes (***n=***1482)	***P-***value
		n (%)	n (%)		n (%)	n (%)	
**Sex**							
Male	1106 (45.8)	358 (32.4)	748 (67.6)	0.306	468 (42.3)	638 (57.7)	0.001
Female	1308 (54.2)	398 (30.4)	910 (69.6)		464 (35.5)	844 (64.5)	
**Age (years)**							
19–44	343 (14.2)	182 (53.1)	161 (46.9)	<0.001	234 (68.2)	109 (31.8)	<0.001
45–64	903 (37.4)	202 (22.4)	701 (77.6)		262 (29.0)	641 (71.0)	
≥ 65	1168 (48.4)	372 (31.8)	796 (68.2)		436 (37.3)	732 (62.7)	
**Marital status**							
Single	329 (13.6)	187 (56.8)	142 (43.2)	<0.001	251 (76.3)	78 (23.7)	<0.001
Married	1721 (71.3)	419 (24.3)	1302 (75.7)		506 (29.4)	1215 (70.6)	
Other (divorced, widowed, separated)	364 (15.1)	150 (41.2)	214 (58.8)		175 (48.1)	189 (51.9)	
**Area of residence**							
Capital area	693 (28.7)	509 (29.6)	1212 (70.4)	0.004	622 (36.1)	1099 (63.9)	<0.001
Non-capital area	1721 (71.3)	247 (35.6)	446 (64.4)		310 (44.7)	383 (55.3)	
**Education level**							
≤ Elementary school graduate	981 (40.6)	318 (32.4)	663 (67.6)	0.001	374 (38.1)	607 (61.9)	0.050
Middle school graduate	305 (12.6)	74 (24.3)	231 (75.7)		98 (32.1)	207 (67.9)	
High school graduate	677 (28.0)	241 (35.6)	436 (64.4)		278 (41.1)	399 (58.9)	
≥ College graduate	451 (18.7)	123 (27.3)	328 (72.7)		182 (40.4)	269 (59.6)	
**Occupation category**							
White collar	265 (11.0)	58 (21.9)	207 (78.1)	<0.001	98 (37.0)	167 (63.0)	<0.001
Sales and service Associates	249 (10.3)	67 (26.9)	182 (73.1)		84 (33.7)	165 (66.3)	
Blue collar	685 (28.4)	169 (24.7)	516 (75.3)		223 (32.6)	462 (67.4)	
Other (students, housewives, unemployed)	1215 (50.3)	462 (38.0)	62.0 (62.0)		527 (43.4)	688 (56.6)	
**Income level (million KRW)**							
< 2	1267 (52.5)	427 (33.7)	840 (66.3)	0.029	493 (38.9)	774 (61.1)	0.830
2–4	646 (26.8)	186 (28.8)	460 (71.2)		243 (37.6)	403 (62.4)	
> 4	501 (20.8)	143 (28.5)	358 (71.5)		196 (39.1)	305 (60.9)	
**Subjective health status**							
Good	599 (24.8)	187 (31.2)	412 (68.8)	0.061	246 (41.1)	353 (58.9)	0.072
Moderate	888 (36.8)	255 (28.7)	633 (71.3)		317 (35.7)	571 (64.3)	
Poor	927 (38.4)	314 (33.9)	613 (66.1)		369 (39.8)	558 (60.2)	
**Subjective stress level**							
Very high	173 (7.2)	60 (34.7)	113 (65.3)	0.303	78 (45.1)	95 (54.9)	0.137
High	662 (27.4)	208 (31.4)	454 (68.6)		245 (37.0)	417 (63.0)	
Low	1058 (43.8)	313 (29.6)	745 (70.4)		396 (37.4)	662 (62.6)	
Very low	521 (21.6)	175 (33.6)	346 (66.4)		213 (40.9)	308 (59.1)	
**Symptoms of depression**							
No	788 (32.6)	198 (25.1)	590 (74.9)	<0.001	273 (34.6)	515 (65.4)	0.005
Yes	1626 (67.4)	558 (34.4)	1068 (65.7)		659 (40.5)	967 (59.5)	
**Chronic disease**							
No	937 (38.8)	347 (37.0)	590 (63.0)	<0.001	432 (46.1)	505 (53.9)	<0.001
Yes	1477 (61.2)	409 (27.7)	1658 (72.3)		500 (33.9)	977 (66.1)	

### Factors associated with health check-up and cancer screening participation among family caregivers of patients with dementia

Table 3 shows the results of the binomial logistic regression analysis to identify factors associated with health check-up and cancer screening participation. Health check-up participation was higher among females (odds ratio [OR]=1.30) than among males. Based on age, the OR for the group aged 19–44 years was lower than that for the other age groups (aged 45–64 years OR=2.70, aged ≥ 65 years OR=2.49). According to marital status, the group of married individuals and those with other marital statuses (divorced, widowed, separated) showed higher participation than single individuals (married OR=3.22; other marital status OR=1.63). According to chronic disease status, health check-up participation was higher in the group who had one or more chronic diseases (OR=1.47). Additionally, for education level, health check-up participation was lower among those who were ≤ elementary school graduates and high school graduates compared to those who were ≥ college graduates (≤ elementary school graduates OR=0.60, high school graduates OR=0.63). For occupation category, sales and service associates, blue-collar workers, and others (students, housewives, and unemployed) reported lower participation in health check-ups compared to white-collar workers (sales and service associates OR=0.58, blue-collar OR=0.56, other OR=0.31). Furthermore, those with symptoms of depression (OR=0.67) showed lower participation than those without symptoms of depression.

**Table 3:** Factors associated with health check-up and cancer screening participation among family caregivers of patients with dementia

Variable	Health check-ups		Cancer screening	
	OR	95% CI	OR	95% CI
**Sex**				
Male^a^	1.00		1.00	
Female	1.30^*^	1.06–1.60	1.61^***^	1.32–1.97
**Age**				
19–44 years^a^	1.00		1.00	
45–64 years	2.70^***^	1.89–3.86	2.77^***^	1.95–3.95
≥ 65 years	2.49^***^	1.59–3.90	2.56^***^	1.65–3.97
**Marital status**				
Single^a^	1.00		1.00	
Married	3.22^***^	2.27–4.57	6.08^***^	4.25–8.69
Other (divorced, widowed, separated)	1.63^*^	1.09–2.45	2.87^***^	1.90–4.32
**Area of residence**				
Capital area^a^	1.00		1.00	
Non-capital area	0.82	0.67–1.01	0.80^*^	0.65–0.98
**Education level**				
≥ College graduate^a^	1.00		1.00	
High school graduate	0.63^**^	0.46–0.85	0.79	0.58–1.07
Middle school graduate	0.79	0.53–1.18	0.74	0.51–1.08
≤ Elementary school graduate	0.60^**^	0.42–0.86	0.59^**^	0.42–0.84
**Occupation category**				
White collar^a^	1.00		1.00	
Sales and service Associates	0.58^*^	0.36–0.93	0.76	0.49–1.19
Blue collar	0.56^**^	0.37–0.85	0.71	0.48–1.05
Other (students, housewives, unemployed)	0.31^***^	0.21–0.47	0.46^***^	0.31–0.68
**Income level**				
> 4 million KRW^a^	1.00		1.00	
2–4 million KRW	0.93	0.69–1.24	1.00	0.76–1.32
< 2 million KRW	0.81	0.61–1.07	0.96	0.66–1.19
**Subjective health status**				
Good^a^	1.00		1.00	
Moderate	0.98	0.76–1.28	1.01	0.79–1.32
Poor	0.88	0.65–1.18	0.96	0.72–1.27
**Subjective stress levels**				
Very high^a^	1.00		1.00	
High	0.83	0.55–1.25	0.98	0.66–1.46
Low	1.05	0.72–1.53	1.28	0.89–1.85
Very low	1.06	0.72–1.54	1.36	0.94–1.97
**Symptoms of depression**				
No^a^	1.00		1.00	
Yes	0.67^***^	0.54–0.82	0.65^***^	0.53–0.80
**Chronic disease**				
No^a^	1.00		1.00	
Yes	1.38^**^	1.10–1.74	1.25	1.00–1.56

*OR* odds ratio, *CI* confidence interval

^a^Reference category

^*^P < 0.05, ^**^ *P* < 0.01, ^***^ *P* < 0.001

Cancer screening participation was higher among women (OR=1.61). Additionally, the OR for the group aged 19–44 years was lower than that for the other age groups (aged 45–64 years OR=2.77, aged ≥ 65 years OR=2.56). Married individuals and individuals in other marital status groups (divorced, widowed, separated) showed higher levels of participation than those who were single (married OR=6.08, other OR=2.87). Concerning the area of residence, the cancer screening rate was higher for capital area residents than for non-capital area residents (OR=0.80). Based on education level, cancer screening participation was lower among elementary school graduates than those with at least a college education (≤ elementary school education OR=0.59). For the occupation category, other occupation groups (students, housewives, unemployed) reported lower cancer screening participation compared to white-collar workers (other occupation groups OR=0.46). Finally, those with symptoms of depression (OR=0.65) showed lower levels of participation than those without symptoms of depression.

## Discussion

This cross-sectional study identified factors associated with health check-up and cancer screening participation among family caregivers of patients with dementia. We found that the health check-up and cancer screening rates among family caregivers of patients with dementia were lower than those among individuals who were not caregivers of patients with dementia. This finding is consistent with our assumption that family caregivers of patients with dementia may not have spare time for health check-ups or cancer screening because of their informal care burden and consequent burnout [6]. Caregivers are hidden patients themselves [9, 10]. Their role as caregivers can have serious adverse physical and mental health consequences since being a caregiver is physically and emotionally demanding and may result in them neglecting their own health and self-care behaviours [29]. Considering that family caregivers may experience various health issues [9, 30, 31], not actively participating in health check-ups and cancer screenings may cause existing conditions to worsen and defer timely disease treatment and management [32]. Indeed, the needs of the care recipients are prioritised over the needs of the family caregivers [6]. This finding emphasises the need to proactively approach family caregivers as clients and potential patients in need of support at the national level.

Among the factors associated with health check-ups and cancer screening, most are consistent with previous studies; however, some of our findings are notable. In terms of demographic factors, health check-up and cancer screening rates were higher among females than males. This difference may be influenced by the lack of available time men may have to participate in health check-ups, because they traditionally perform more social and economic activities than women in Korea [25, 29]. Furthermore, an important obstacle is the apparent reluctance men have to consult a doctor [33, 34]. Consequently, men often do not seek help until a disease has progressed [35]. Therefore, sex is an important factor in determining health behaviours and should be considered to increase screening participation [33].

Additionally, older people showed a higher health rate of participation in check-ups, which was consistent with previous findings that have indicated that non-participation in cancer screening and general examinations is higher among relatively younger people [25]. In Korea, the fact that people aged < 40 years are not eligible for the national screening and must undergo private screening may explain these results [36].

Furthermore, the health check-up rate was higher among married individuals and those with other marital statuses compared to that among single adults. This result supports a previous finding that married people show relatively better health check-up behaviours [23], which may be attributable to encouragement to undergo health check-ups from the spouse, suggesting that encouragement and support are needed to increase health check-up participation [37, 38].

In terms of educational level, the health check-up rate was lower among ≤ elementary school, middle school, and high school graduates compared to that among ≥ college graduates; in addition, the cancer screening rate decreased with decreasing educational level. This could be explained by a previous finding that individuals with at least a college education voluntarily undergo health check-ups because they are sufficiently aware of their benefits [39]. Furthermore, because education level is closely related to health behaviours, attitudes, and knowledge in general, people with higher education levels undergo health check-ups in an effort to improve their lifestyles and continue living a healthy life [40]. Since caregivers with less education may lack access to healthcare and caregiver support, interventions need to aim to increase the capability of this population to identify and understand comprehensively their unmet health needs and access support more easily [36, 41]. Therefore, healthcare researchers and policymakers should be aware of these marginalised groups and consider their needs when developing policy goals and support for family caregivers of patients with dementia [41].

Moreover, sales and service associates, blue-collar workers, and others (students, housewives, and unemployed) showed lower health check-up rates than white-collar workers. Furthermore, sales and service associates and blue-collar workers also had lower cancer screening rates than white-collar workers. These results reflect previous findings showing that health check-up rates are higher among office workers [42].

In terms of mental health status, health check-up and cancer screening rates were found to be lower in people with symptoms of depression. We speculate that people with stress and depression also experience demotivation and reduced activity, potentially hindering their participation in health check-ups [43]. Depression is often associated with the use of health services; however, the prevalence of depression in family caregivers of patients with dementia is high. Therefore, this factor must be considered when supporting family caregivers of patients with dementia [44]. In other words, attention should be paid to psychological factors, such as depression, and strategies should be developed to increase the health check-up and cancer screening rates in family caregivers of patients with dementia [8–10]. Furthermore, health check-up participation rates were higher among family caregivers with chronic diseases. Family caregivers who are diagnosed with a chronic condition may need to pay more attention to their health management [22]. According to previous studies, chronic diseases are highly correlated with perceived health conditions and check-up participation; if a chronic disease is present, interest in health care increases, which may be associated with increased check-up participation [45, 46]

This study had several limitations. First, the cross-sectional design of our study did not allow us to examine causal relationships over time. Second, because we used secondary data for our analysis, the responses of some participants could not be examined in further detail. Although participation must be considered according to the type of health check-up and cancer screening, these data collected all types of participation without distinguishing them by type. In addition, the health check-up and cancer screening participation of caregivers of patients with chronic diseases or cancer should have been compared with caregivers of patients with dementia to examine the health check-up and cancer screening participation rates. However, this comparison could not be made because of the data limitations. Thus, further studies should consider various types of check-ups and screenings. In addition, studies should examine factors of caregivers’ check-up or screening participation in relation to various diseases.

Despite these limitations, the significance of this study lies in providing baseline data and implications for improving existing policies and developing new policies to increase health check-up and cancer screening participation in family caregivers of patients with dementia. This was achieved by filling a gap in the literature on health check-up rates in family caregivers of patients with dementia in Korea by analysing factors associated with individuals’ health check-up and cancer screening participation.

## Conclusions

To ensure a healthy life for families providing care, checking the patient’s health routinely to detect and treat abnormalities early and prevent them from developing further complications is crucial. This study revealed that the physical and mental health of family caregivers of patients with dementia are closely associated with health check-up and cancer screening participation. The results of this study emphasise the need to proactively approach family caregivers as clients or potential patients who need support at the governmental level. For instance, doctors and nurses at primary medical care centres should educate family caregivers as part of the treatment plan for patients with dementia. In addition, this study found that family caregivers with depressive symptoms and lower education levels were less likely to participate in check-ups and screenings. This limitation necessitates the development of more targeted education and management strategies. Promoting the physical and mental health of people directly involved in the care of patients with dementia warrants further research to address the current lack of information on this population’s health screening behaviours.

## Data Availability

The dataset supporting the conclusions of this article is available in the KCHS repository, https://chs.kdca.go.kr/chs/index.do

## Abbreviations

KCHS: Korea Community Health Survey
KCDC: Korea Centres for Disease Control and Prevention
KRW: Korean Won
PHQ-9: Patient Health Questionnaire-9
OR: Odds ratio
CI: confidence interval
